# Synthesis of Novel 4-[1-(4-fluorobenzyl)-5-imidazolyl] Dihydropyridines and Studying their Effects on Rat Blood Pressure 

**Published:** 2011

**Authors:** Seyed Ahmad Mohajeri, Hossein Hosseinzadeh, Sara Salami, Vahidehsadat Motamedshariaty, Mahmoud Seifi, Farzin Hadizadeh

**Affiliations:** 1 * Pharmaceutical Research Center, School of Pharmacy, Mashhad University of Medical Sciences, Mashhad, Iran*; 2 * Biotechnology Research Center, School of Pharmacy, Mashhad University of Medical Sciences, Mashhad, Iran*

**Keywords:** Blood pressure, Calcium Channel Blockers, Heart rate, Rats

## Abstract

**Objective(s):**

Calcium-channel blockers have an important role in the treatment of several cardiovascular disorders. Derivatives of 1, 4-dihydropyridine are one of the most potent calcium antagonists. In this study four novel 1, 4-dihydropyridine calcium channel blockers were synthesized and their hypotensive properties were investigated in male rats.

**Materials and Methods:**

Four 1, 4-dihydropyridines bearing 1-(4-fluorobenzyl)-5-imidazolyl substituent at 4 position (**5a-d**) were synthesized and tested for hypotensive activity in male rats. The animal was anaesthetized and the right jugular vein was cannulated for the administration of test agents. The left carotid artery was cannulated and connected to a pressure transducer for continuous monitoring of arterial blood pressure.

**Results:**

All synthesized compounds lowered rat blood pressure significantly in comparison with DMSO as vehicle and nifedipine as positive control. The hypotensive effects of all compounds were less than that of nifedipine at 2 and 4 mg/kg (*P*< 0.05). The order of their effects on mean arterial blood pressure (MABP) was **5b**>**5c**>**5a**>**5d** at dose of 4 mg/kg (*P*< 0.05). All compounds tested increased heart rate in comparison with DMSO (*P*< 0.05). The chronotropic effect of nifedipine was significantly less than synthesized compounds at dose of 4 mg/kg (*P*< 0.01).

**Conclusion:**

The results showed that these novels 1, 4-dihydropyridines decreased mean arterial blood pressure (MABP) significantly, while increased heart rate in rat.

## Introduction

Calcium-channel blockers have an important role in the treatment of several cardiovascular disorders (1-3). They have been widely used for hypertension, angina pectoris, heart failure and Raynaud’s disease (4,5). Structurally diverse group of compounds are known to be effective as calcium antagonists (6). The most potent class of antagonists comprises derivatives of 1,4-dihydropyridine of which amlodipine is used widely today (7,8). Dihydropyridines act by inhibiting the influx of calcium ion into the vascular smooth muscle cells via L-type calcium channels (9,10). Their useful effects in management of cardiovascular disorders are due to their ability to relax vascular smooth muscles. In angina pectoris, such drugs decrease the resistance in systemic and coronary arterial beds, thereby reducing cardiac oxygen requirement and increasing cardiac oxygen supply, respectively (10). 

This class of compounds has been the subject of many structure-activity relationship (SAR) studies (6, 11-14). Developments in the chemistry of dihydropyridines have been reviewed (15). Synthesis of compounds with greater tissue selectivity, longer duration of action and slower rate of absorption have been the main aim of such efforts (2).

**Figure 1.**
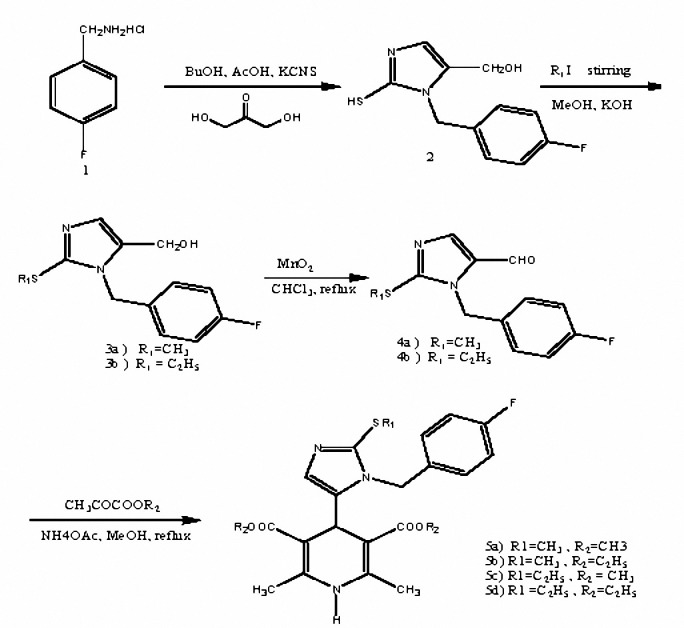
Schematic representation for synthesis of compounds 5a-d.

In our previous studies, some new 1,4-dihydropyridines were synthesized and their antihypertensive properties were reported (8,16). In this work, some novel 4-[1-(4-fluorobenzyl)-5-imidazolyl] dihydropyridines **(5a-d)** were synthesized (Figure 1) and their hypotensive effects on rat blood pressure and heart rate were studied. Our aim was to study the effects of these compounds in comparison with that of nifedipine as a standard 1,4-dihydropyridine.

## Materials and Methods

***General instruments and chemicals***

Melting points were determined using the capillary apparatus with a system of Gallenkamp. ^1^H-NMR spectra were run on a Bruker AC-80 spectrometer. Infrared spectra were recorded on a FT-IR Perkin- Elmer Paragon 1000 spectro-photometer. 

Nifedipine was purchased from Tolidarou Pharmaceuticals (). Xylazine and ketamine were obtained from Alfasan Co, . All compounds including nifedipine were dissolved in dimethyl sulfoxide (DMSO). Other analytical grade reagents were obtained from Merck Company (). 

***Chemistry***

Symmetrical dihydropyridines (**5a-d)** were synthesized by classical Hantzch condensation as described previously (16). 

*Preparation of 1-(4-fluorobenzyl)-5-hydroxymethyl-2-thio-imidazoles (2)*

A suspension of 4-fluorobenzylamine hydrochloride **1 **(100 mmol), potassium thiocyanate (10.35 g, 100 mmol) and dihydroxyacetone (6.4 g, 701mmol) in glacial acetic acid (8 ml) and 1-butanol (50 ml) was stirred for 70 hr. After adding water (10 ml), the resulting mixture was filtered. The precipitate was washed with water (30 ml) and diethyl ether (30 ml), respectively to give the compounds **2,** yield 65%; mp 186-189°C; IR (KBr): 3112 cm^-1^(OH); ^1^H NMR (DMSO-d_6_):11.8 (bs, 1H, SH), 7.5-7.1 (2d, 4H, H-Ar), 6.59 (s, 1H, H-imidazole), 5.06 (s, 2H, CH_2_N), 3.91 (s, 2H, CH_2_O).

*General procedures for preparation of 1-(4-fluorobenzyl)-2-alkylthio-5-hydroxymethylimidazoles (3a, b)*

To a stirred suspension of **2a** (22.72 mmoles) in methanol (350 ml) sodium hydroxide (1 N, 24 ml) was added at room temperature. The resulting mixture was stirred for 10 min until a clear pale yellow solution was obtained. Appropriate alkyl iodide (23.9 mmoles) was then added dropwise. Then, the solution was stirred overnight. After concentrating the solvent at reduced pressure, water (200 ml) was added to the residue and extracted with chloroform (370 ml). The chloroform was evaporated to give the corresponding compounds **3a, b**.

***1-(4-Fluorobenzyl) - 5-hydroxymethyl-2-methylthio-imidazole (3a)***

Yield 76%; mp 140-142°C; IR (KBr): 3200 cm^-1^(OH); ^1^H NMR (CDCl_3_): 7.38-6.99 (m, 5H, Ar-H, H-Imidazole), 5.28 (s, 2H, CH_2_N), 4.45(s, 2H, CH_2_O), 3.5 (s, ), 2.5 (s, 3H, CH_3_S).

*2-Ethylthio-1-(4-fluorobenzyl)-5-hydroxymethylimidazole (3b)*

Yield 78%; mp 116-119°C; IR (KBr):3200 cm^-1^(OH); ^1^H NMR (CDCl_3_): 7.60-7.00 (m, 5H, Ar-H, H-imidazole), 5.58 (s, 2H, CH_2_N), 4.60 (s, 2H, CH_2_O), 3.07 (q, 2H, CH_2_S, J= 8 Hz), 1.39 (t, 3H, CH_3_S, J= 8 Hz).

*Preparation of formylimidazoles (4a, b)*

A stirring suspension of **3a, b** (4.27 mmoles) and manganese dioxide (2.4 g, 27.6 mmoles) in chloroform (50 ml) was refluxed overnight. The reaction mixture was cooled to room temperature and filtered on diatomatceous earth. The chloroform was evaporated at reduced pressure to give the corresponding aldehydes **4a, b**.

*5-Formyl-1-(4-fluorobenzyl)-2-methylthio-imidazole (4a)*

Yield 80%; mp 84-86°C; IR (KBr): 1660 cm^-1^ (C=O); ^1^H-NMR (CDCl_3_): 9.6 (s, 1H, CHO), 7.76 (s, 1H, H-imidazole), 7.46-7.04 (m, 4H, Ar-H), 5.49 (s, 2H, CH_2_N), 2.68 (s, 3H, CH_3_S).

*2-Ethylthio-5-formyl-1-(4-fluorobenzyl) imidazole (4b)*

Yield 80%; mp 55.5-57.5 °C; IR (KBr):1661 cm^-1^(C=O); ^1^H NMR (CDCl_3_): d 9.6 (s, 1H, CHO), 7.78 (s, 1H, H-imidazole), 7.46 -7.04 (m, 4H, Ar-H), 5.49 (s, 2H, CH_2_N), 3.27(q, 2H, CH_2_S, J=8 Hz), 1.39(t, 3H, CH_3_S, J=8 Hz).

*General procedure for preparation of dialkyl 1,4-dihydro-2,6-dimethyl-4-[1-(4-fluorobenzyl)-2-alkylthio-5-imidazolyl]-3,5-pyridinedicarboxylate (5a-d)*

A solution of ammonium hydroxide (25%, 0.5 ml) was added to a stirring solution of compound **4** (1.26 mmol) and alkyl acetoacetate (2.54 mmol) in methanol (5 ml). The mixture was protected from light and refluxed overnight. The methanol was evaporated at reduced pressure to give compounds **5a-d**. Spectral data of compounds **(5a-d**) were given in Table 1. 

***Pharmacology***

*Experimental design*

Animals were divided into 6 groups (n= 5, in each group), and assigned to receive dimethylsulfoxide (DMSO, 0.04 ml) as vehicle, nifedipine, **5a**, **5b**, **5c** and **5d** at 1, 2 and 4 mg/kg.

*Experimental protocol*

This study was carried out on male Wistar rats (Razi Institutes, Mashhad, Iran) weighing between 200 and 250 g. Rats were housed 5 per cage with a 12/12 hr light/dark cycle at 21±2 °C and had free access to food and water in animal room of Pharmaceutical Research Center, Mashhad University of Medical Sciences. Animals were anaesthetized with intraperitoneal injections of 6 mg/kg xylazine (Alfasan, ) and 60 mg/kg ketamine (Alfasan, ). The right jugular vein was cannulated for the administration of test agents (**5a-d**), nifedipine and vehicle throughout the experiment. The left carotid artery was cannulated with a cannula containing heparinized saline (50 IU/ml) and connected to a pressure transducer (SP 844, Capto) for continuous monitoring of arterial blood pressure. Acquisition data were performed by a PowerLab 4/30 (). Data processing and analyzing were carried out by Chart^TM^ 5 version 5.5.6 software (). The trachea was cannulated and the animals were allowed to breathe spontaneously. Body temperature was recorded using a rectal thermostat probe and was maintained at 37±0.5 °C using an incandescent lamp placed over the abdomen. After stabilization, arterial blood pressure (systolic, diastolic and mean), and heart rate were recorded. 

*Calculations and statistical analysis*

MABP was calculated as two third of diastolic pressure plus one third of systolic pressure (10). The changes in mean arterial pressure and heart rate were calculated as the percentages of their values prior to administration of vehicle or drugs. The data, presented as mean±SEM, were analyzed using one way analysis of variance (ANOVA) followed by a Tukey-Kramer multiple comparison test (for comparison of dihydropyridines effects with DMSO in rats). *P* value of less than 0.05 was considered to be significant.

## Results

***Chemistry***

1-(4-Fluorobenzyl)-5-hydroxymethyl-2-thio-imidazole (**2a, b**) was prepared from 4-fluorobenzylamine hydrochloride (**1**) and dihydroxyacetone dimmer. Reaction of **2** (Figure 1) with alkyl halide afforded corresponding 2-alkylthio-1-(4-fluorobenzyl)-5-hydroxymethylimidazole (**3a, b**). Oxidation of **3** with manganese dioxide in chloroform gave corresponding aldehyde (**4a, b**). The symmetrical 1,4-dihydropyridines (**5a-d**) were prepared by the classical Hantzsch condensation in which the aldehyde (**4a, b**) was reacted with acetoacetic acid ester and ammonium hydroxide. Spectral data of compounds (**5a-d**) were given in Table 1.

***Pharmacology***

Administration of DMSO (0.04 ml) as vehicle caused a decrease in MABP (5.05%0.69) and increase in heart rate (1.66%0.54) compared to their values before DMSO administration.

**Figure 2.**
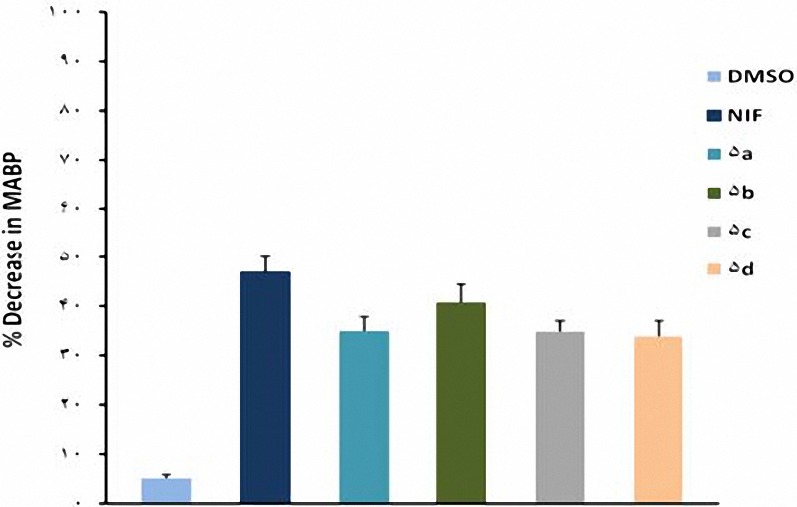
Decrease in mean arterial pressure (Mean±SEM) by the administration of DMSO (0.04 ml), nifedipine (1 mg/kg) and compounds 5a-d (1 mg/kg). ^***^P< 0.001, compared with DMSO as control, ^+^* P*< 0.05, ^++^* P*< 0.01, compared with nifedipine (n= 5).

**Figure 3.**
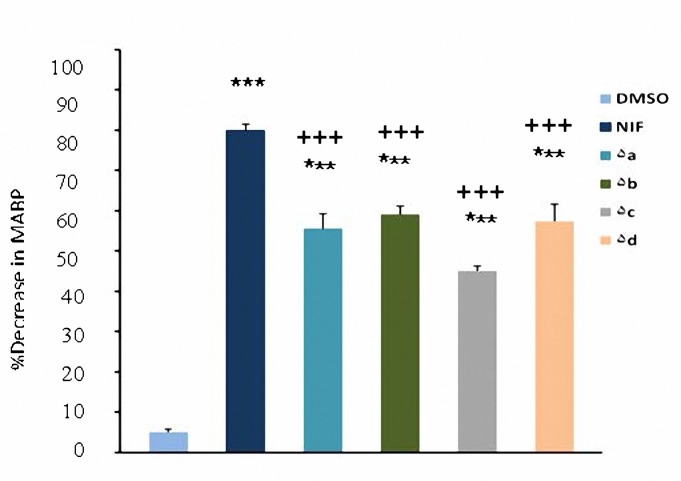
Decrease in mean arterial pressure (mean±SEM) by the administration of DMSO (0.04 ml), nifedipine (2 mg/kg) and compounds 5a-d (2 mg/kg). ***P< 0.001, compared with DMSO as control,^ ++^*P*  0.01,^ +++^*P*  0.001, compared with nifedipine (n= 5).

**Figure 4.**
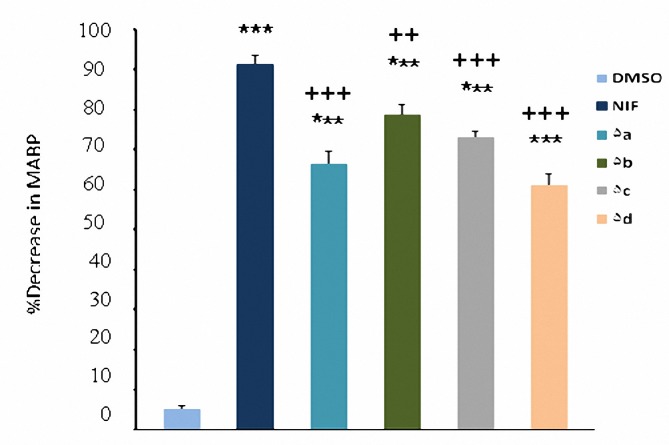
Decrease in mean arterial pressure (mean±SEM) by the administration of DMSO (0.04 ml), nifedipine (4 mg/kg) and compounds 5a-d (4 mg/kg). ***P< 0.001, compared with DMSO as control, ^++^*P*< 0.01,^ +++^*P*< 0.001, compared with nifedipine (n= 5).

In comparison with DMSO (0.04 ml), nifedipine (1, 2 and 4 mg/kg) and compounds **5a-d** (1, 2 and 4 mg/kg ) caused significant (*P*< 0.05) decrease in MABP (Figure 2-4). The hypotensive effects of all compounds were less than that of nifedipine at 2 and 4 mg/kg (*P*< 0.01). No significant difference was seen between **5b** and nifedipine at 1 mg/kg (*P*> 0.05) whereas other compounds had less effects in lowering MABP in comparison with nifedipine (*P*< 0.05) at the same dose. At dose of 4 mg/kg hypertensive effects caused by **5a** was significantly more than **5b** (*P*< 0.05); and also hypotensive effect of **5b** was more than **5d** (*P*< 0.001). It was also found that **5c** was more potent than **5d** (*P*< 0.05).

**Figure 5.**
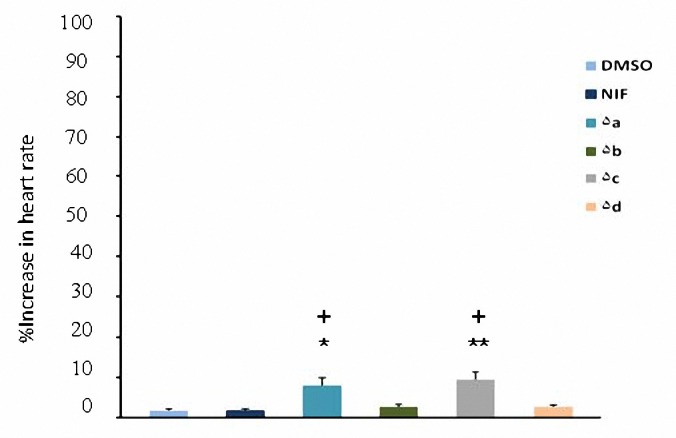
Increase in heart rate (mean±SEM) by the administration of DMSO (0.04 ml), nifedipine (1 mg/kg) and compounds 5a-d (1 mg/kg). *P< 0.05, **P< 0.01, compared with DMSO as control, ^+^* P*< 0.05, compared with nifedipine (n= 5).

**Figure 6.**
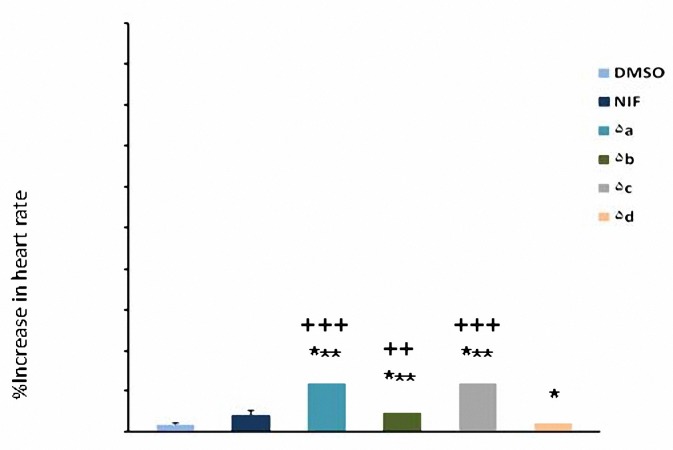
Increase in heart rate (mean±SEM) by the administration of DMSO (0.04 ml), nifedipine (2 mg/kg) and compounds 5a-d (2 mg/kg). *P< 0.05, ***P< 0.001, compared with DMSO as control, ^++^*P*<0.01, ^+++^*P*< 0.001, compared with nifedipine (n= 5).

**Figure 7.**
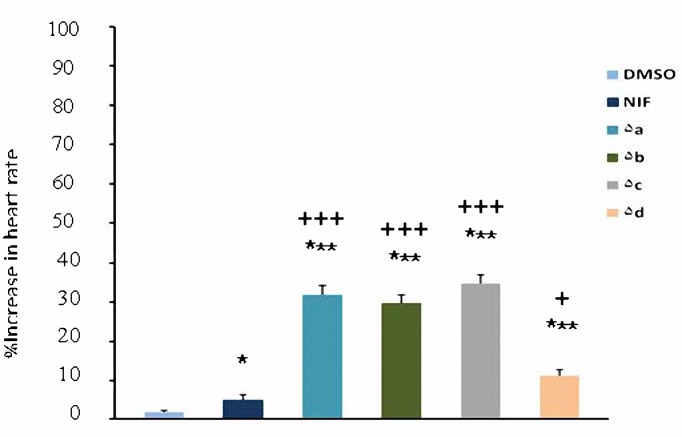
Increase in heart rate (mean±SEM) by the administration of DMSO (0.04 ml), nifedipine (4 mg/kg) and compounds 5a-d (4 mg/kg). *P< 0.05, *** P< 0.00, compared with DMSO as control, ^+^*P*< 0.05,^ +++^* P*< 0.001, compared with nifedipine (n= 5).

All test compounds (**5a-d**) increased heart rate in comparison with DMSO at 2 and 4 mg/kg (*P*< 0.05) but at 1 mg/kg only compounds **5a** and **5c** had significant effect (*P*< 0.05). Increased heart rate caused by nifidipine was significant only at 4 mg/kg. The chronotropic effect of nifedipine was significantly less than that of synthesized compounds (*P*< 0.01) at dose of 4 mg/kg (Figures 5-7). At dose of 2 mg/kg increased heart rate caused by nifedipine was significantly less than **5a**, **5b** and **5c** (*P*< 0.05) whereas there was not any significant difference with **5d **(*P*> 0.05).

## Discussion

The present study demonstrates that all four tested analogues of nifedipine exert discriminatory effects on MABP and heart rate. These effects were significantly different than that of DMSO as solvent. Their effects in lowering MABP were significantly less than that of nifedipine except **5b** at 1 mg/kg. The mechanism of the hypotensive effect of dihydropyridines **5a-d** could not be established in the present study, but the immediate reduction in blood pressure suggests that blood vessels contractility is affected by these compounds. A potent relaxant effect of similar dihydropyridines on smooth muscles of rat colon via calcium channel blocking effect has been previously reported (17). The results of another study have suggested a potent inhibitory effect of dihydropyridines on guinea-pig ileum smooth muscle (18). Therefore, it seems that blocking of calcium channels may contribute to blood vessels relaxation and hypotensive effects.

Compounds **5a-d** increased heart rate significantly, compared to DMSO, at 2 and 4 mg/kg (p<0.05). This effect could be the result of vasodilatory effects of test compounds. The reflex tachycardia has been previously reported for classic dihydropyridine compounds like nifedipine (19). Reflex tachycardia for some new dihydropyridines was also reported by Miri *et a. *in another study (10). The effects of similar dihydropyridines on isolated right atrium in rats has also been reported previously (17). 

From the SAR viewpoint, comparing compound **5b** (R_1_=CH_3_, R_2_=C_2_H_5_) with **5d** (R_1_=C_2_H_5_, R_2_= C_2_H_5_) showed that methyl substituent is better tolerated at R_1_ position. This may be due to steric factor at this position in binding to calcium channels. On the other hand, comparing to **5a** (R_1_=CH_3_, R_2_=CH_3_), higher hypotensive effect of **5b** (R_1_=CH_3_, R_2_=C_2_H_5_) indicated that ethyl substituent gives superior lipophilic calcium binding activity to dihydropyridine compound. This result was in agreement with previous SAR of dihydropyridines (6). 

## Conclusion

In this work, a series of four new dihydropyridines (**5a-d**) were synthesized and their effects on blood pressure and heart rate were studied. These compounds decreased MABP significantly, while increased heart rate in rats. Further pharmacological and toxicological studies are required to provide a comprehensive profile of these compounds for their prospective use in drug therapy.
